# Effects of Multicomponent Versus Aerobic Training on Body Composition, Physical Fitness, Psychological Health, and Quality of Life in Cancer Survivors: A 24-Week Randomized Controlled Trial

**DOI:** 10.3390/sports14040135

**Published:** 2026-04-01

**Authors:** Alessandro Petrelli, Ilaria Pepe, Luca Poli, Gianpiero Greco, Carla Minoia, Antonella Daniele, Patrizia Dicillo, Francesca Romito, Francesco Fischetti, Stefania Cataldi

**Affiliations:** 1 Department of Translational Biomedicine and Neuroscience (DiBraiN), University of Study of Bari, 70124 Bari, Italy; alessandro.petrelli@uniba.it (A.P.); ilaria.pepe@uniba.it (I.P.); francesco.fischetti@uniba.it (F.F.); 2 Department of Neurosciences, Biomedicine and Movement Sciences, University of Verona, 37129 Verona, Italy; 3 Hematology Unit, IRCCS Istituto Tumori “Giovanni Paolo II”, 70124 Bari, Italy; c.minoia@oncologico.bari.it; 4 Pathology Anatomy—Clinical Nutrition Unit, IRCCS Istituto Tumori “Giovanni Paolo II”, 70124 Bari, Italy; antonella.daniele@oncologico.bari.it; 5 Rehabilitation Service, IRCCS Istituto Tumori “Giovanni Paolo II”, 70124 Bari, Italy; p.dicillo@oncologico.bari.it; 6 Psycho-Oncology Service, IRCCS Istituto Tumori “Giovanni Paolo II”, 70124 Bari, Italy; f.romito@oncologico.bari.it; 7 Department of Education and Sport Sciences, Pegaso Telematic University, 80143 Naples, Italy; stefania.cataldi@unipegaso.it

**Keywords:** cancer, physical activity, resistance exercise, aerobic exercise, lean body mass, functional fitness, psychological well-being

## Abstract

Background: Cancer survivors frequently experience persistent physical and psychological sequelae, including impaired physical function, fatigue, anxiety/depressive symptoms, and reduced health-related quality of life (HRQoL). Exercise is an effective non-pharmacological intervention; however, comparative evidence between multicomponent training (MCT) and aerobic training (AT) using a multidomain framework remains limited. Methods: In this randomized controlled parallel-group trial, 47 cancer survivors (mean age 63.0 ± 8.9 years) were allocated to a 24-week supervised MCT programme (n = 16), an AT programme (n = 16), or a non-exercise control group (CG; n = 15). Outcomes were assessed at baseline and post-intervention including body composition (BIA), physical performance, fatigue (FSS), anxiety (STAI-Y1/Y2), depressive symptoms (BDI), and HRQoL (EORTC QLQ-C30). Results: Fat mass decreased in both MCT (*p* = 0.005) and AT (*p* = 0.034), whereas arm circumference increased only in MCT (*p* < 0.001). Significant Group × Time interactions were observed for major physical performance outcomes; improvements were broader in MCT, while AT showed its largest change in aerobic endurance. Between-group contrasts indicated greater gains with MCT than AT for chair-stand (*p* = 0.046), sit-and-reach (*p* = 0.048), and handgrip strength (*p* = 0.049). Significant interaction effects were also observed for fatigue and psychological outcomes (FSS: *p* = 0.003; STAI-Y1 and STAI-Y2: *p* < 0.001; BDI: *p* < 0.001) and for HRQoL global health (*p* = 0.003), with larger improvements in MCT than AT for fatigue, state anxiety, and depressive symptoms (all *p* < 0.05), but not for trait anxiety (*p* > 0.05). Conclusions: A 24-week supervised MCT programme produced broader benefits than AT alone across physical function and selected psychological outcomes in cancer survivors. These findings support the incorporation of multicomponent exercise into survivorship care as a feasible and effective strategy for addressing multidimensional treatment sequelae.

## 1. Introduction

Cancer survivorship has increased substantially in recent decades due to advances in early detection and treatment [1,2]. According to the National Cancer Institute (NCI), a cancer survivor is defined as any individual diagnosed with cancer, from the time of diagnosis throughout the remainder of life, including those living with cancer and those free of cancer [3]. Cancer survivors who have completed primary treatment generally expect to resume daily activities at a level comparable to that prior to diagnosis [4]. However, cancer treatments such as surgery, chemotherapy, radiotherapy, and targeted therapies frequently lead to persistent physical and psychological sequelae that may compromise functional capacity and quality of life.

In many survivors, these treatment-related sequelae extend beyond the acute phase, delaying functional recovery and complicating reintegration into usual life roles [5]. Cancer survivors face unique health challenges as a result of their cancer diagnosis and the impact of treatments on their physical and mental well-being [6]. For example, cancer survivors often experience declines in physical functioning and quality of life [7]. Treatment-related side effects are common and include skeletal muscle atrophy and body composition alterations, as well as reductions in aerobic capacity [8]. Strength and flexibility impairments, fatigue, depressive symptoms, and nausea are also frequently reported [9].

Collectively, these effects contribute to an overall deterioration in quality of life, underscoring survivorship as a period of sustained multidimensional vulnerability [10].

The relationship between body composition and cancer survival has been a subject of study for decades, with special attention paid to the role of adiposity and muscle mass, which have been associated with prognosis across several cancer types [11]. Lean mass undergoes significant decline during and after cancer treatments due to inflammatory and catabolic processes, drug toxicity, symptom burden, reduced intake, and impaired mobility [12,13]; sarcopenia and cachexia can occur even with obesity [14]. Given the established prognostic significance of adiposity and skeletal muscle mass, these body composition parameters were selected as key outcomes in the present study to examine their potential association with survivorship health status and response to the intervention.

Low physical activity and reduced muscle strength are associated with psychological distress and fatigue in cancer survivors [15]. Impairments in cardiorespiratory fitness (CRF) and strength are frequent [16] and are not only related to functional limitations and poorer health-related quality of life (HRQoL) [6], defined as “how well a person functions in their life and their perceived well-being in physical, mental, and social domains of health” [17]. Functioning refers to an individual’s ability to carry out pre-defined activities, whereas well-being reflects subjective perceptions of health, often affected by fatigue and mood disturbances [18]. In addition, CRF and muscle strength have prognostic relevance in oncology, as higher levels are associated with lower cancer-specific and all-cause mortality [19].

Exercise is recognized as one of the most effective non-pharmacological interventions for improving quality of life in cancer survivors [20,21,22,23]. During and after treatment, it improves functional capacity, muscle strength, and mobility while alleviating fatigue and reducing anxiety and depression [24,25]. Moderate-intensity aerobic training is a safe and effective approach to increase endurance [26]. Consistently, recent oncology guidelines recommend integrating structured aerobic and resistance training during and after treatment, with evidence supporting improvements in CRF, strength, fatigue, and patient-reported outcomes [27,28,29].

Aerobic and resistance training have also been associated with improvements in sleep quality, fatigue, mood-related symptoms, and HRQoL [30,31]. These effects have been discussed in relation to broad stress- and recovery-related pathways (e.g., neuroendocrine and immune regulation) [32]. At the outcome level, meta-analytic evidence indicates that aerobic-oriented interventions primarily enhance CRF/endurance and reduce fatigue, whereas resistance exercise produces robust gains in muscle strength and may favourably influence body composition-related parameters [33,34]. Evidence on anxiety and depression is comparatively less consistent, although supervised resistance programs and integrative reviews report reductions in anxiety and depression and improvements in HRQoL, particularly in the post-treatment phase [18,35].

Despite this consolidated evidence, uncertainties remain regarding the optimal exercise modality and the added value of integrating multiple physical components within a single program. In particular, it is still unclear whether an integrated multicomponent program confers advantages over a volume-matched aerobic protocol when outcomes are considered jointly across body composition, physical function, psychological symptoms, and HRQoL. In this regard, multicomponent training (MCT) has gained interest as a time-efficient approach that combines strength, cardiorespiratory endurance, flexibility, and balance within the same session [29,36]. This is because, as defined by Labata-Lezaun et al. [37], MCT is a training protocol that incorporates at least three exercise modalities (such as aerobic training, strength/resistance training, flexibility, balance, and coordination). Although still underexplored in cancer survivorship research [38,39], emerging data in cancer survivors suggest that MCT can improve muscle performance and functional fitness, with concurrent benefits on body composition, fatigue, depression/anxiety, and HRQoL [27,28,38]. However, comparative data testing integrated multicomponent approaches against volume-matched aerobic protocols across multidimensional outcomes remains limited.

To date, direct comparative evidence remains limited regarding MCT and aerobic training (AT) in cancer survivors using a multidimensional and integrative health approach. Therefore, this randomized controlled trial compared a 24-week MCT program versus an equivalent-volume AT program and a control group (CG) on body composition (bioelectrical impedance), functional performance (strength, aerobic capacity, flexibility, mobility), psychological health (fatigue, state/trait anxiety, depressive symptoms), and HRQoL. We hypothesized that participation in structured exercise (MCT or AT) would result in greater pre-to-post improvements than CG across the assessed outcomes. As a secondary expectation, we anticipated larger changes with MCT than AT in outcomes more closely related to resistance and multimodal stimuli, particularly body composition and neuromuscular performance, with corresponding improvements in fatigue and psychological symptom burden.

## 2. Materials and Methods

### 2.1. Research Design

The present study employed a randomized, controlled, parallel-group trial design with two assessment time points (baseline and post-intervention) over a 24-week (six-month) intervention period. Participants were assigned to two structured exercise programs, namely a multicomponent training group (MTG) and an aerobic training group (ATG), both compared with a non-exercise control group (CG). Primary outcome domains included body composition, physical performance, psychological symptoms, and health-related quality of life (HRQoL).

### 2.2. Participants

Participants were recruited from January to July 2025 at the Bari Cancer Institute using a consecutive sampling method. All patients attending routine oncological follow-ups during recruitment were systematically screened for eligibility by trained staff. This approach included a representative sample of cancer survivors, reducing selection bias. Inclusion criteria were: (1) age between 18 and 75 years; (2) having any cancer type confirmed by histology; (3) having completed primary cancer treatment (surgery, chemotherapy, or radiotherapy) at least six months before starting the study; (4) not meeting World Health Organization (WHO) guidelines for physical activity [40], defined as less than 150 min a week of moderate-intensity aerobic activity, less than 75 min a week of vigorous-intensity aerobic activity, or an equivalent combination; and (5) having medical clearance for physical activity from a qualified oncologist or sports medicine doctor.

Exclusion criteria were: (1) metastatic or recurrent cancer; (2) cardiovascular, orthopedic, or systemic conditions that make exercise unsafe; (3) current psychiatric conditions that need intensive treatment or medication adjustment; (4) taking part in any structured physical activity program in the last three months; (5) planning to miss more than two consecutive weeks during the intervention; or (6) being unable to follow the study protocol or complete the required tests.

An a priori power analysis was conducted using G*Power 3.1 to estimate the sample size required to detect a moderate Group × Time interaction (f = 0.25) in the planned mixed-design ANOVA, with α = 0.05 and 80% power. The analysis indicated that a minimum of 42 participants was required. To account for potential dropouts, the recruitment target was set at 51 participants.

Fifty-one eligible participants completed baseline assessments and were randomly allocated (1:1:1) to three groups: MTG (n = 17, multicomponent training, 60 min, 2 sessions/week); ATG (n = 17, aerobic-only training, 60 min, 2 sessions/week); and CG (n = 17, wait-list control, no structured activity during intervention). Participants were randomized to MTG, ATG, or CG using a computer-generated permuted-block sequence (allocation ratio 1:1:1) generated by an independent researcher using Sealed Envelope (permuted-block randomization list generator). Allocation concealment was ensured using sequentially numbered, opaque, sealed envelopes, prepared by an independent researcher not involved in enrollment or assessment, and opened sequentially only after baseline testing. Due to the nature of the intervention, participants and trainers were not blinded; however, outcome assessors and data analysts were blinded to group assignment. During the intervention, four participants withdrew, leaving 47 participants (mean age = 63.04 ± 8.91): MTG (n = 16), ATG (n = 16), and CG (n = 15). All participants maintained prescribed medications, which were not formally monitored. None followed specific diets or received nutritional interventions. All remaining participants completed the study without adverse effects or injuries. Adherence rates: MTG 94.1%, ATG 93.4%.

**Figure 1:**
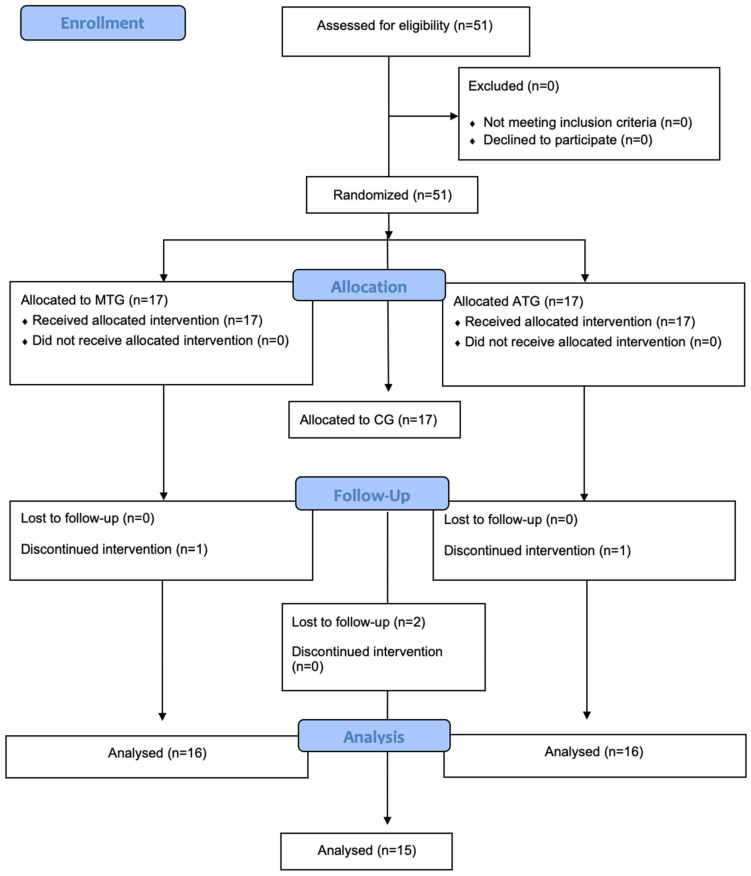
Eligibility flow diagram.

The trial is reported following Consolidated Standards of Reporting Trials (CONSORT) recommendations; participant flow across MTG, ATG, and CG is presented in Figure 1.

Ethics approval was obtained from the Institutional Ethics Committee of the University of Bari Aldo Moro (Bari, Italy) (protocol code 0324886, 30/12/24), in accordance with the Declaration of Helsinki. All participants provided written informed consent. This study was registered at ClinicalTrial.gov (ID: NCT06853613) before the enrolment of participants. The design of the project and the study protocol follow the CONSORT 2025 reporting guidelines [41].

### 2.3. Outcome Measures and Data Collection

Data were collected at baseline (week 1) and after the 24-week (six-month) intervention. Physical performance assessments were conducted at the Angiulli Gymnastics Club (Bari, Italy) in a temperature- and humidity-controlled room (22–23 °C; 40–60% humidity). Testing was scheduled in the morning (8:00–11:00 a.m.) to reduce circadian variability. All procedures were administered by trained staff using standardized protocols.

Body composition was assessed at the Nutrition Outpatient Clinic of the IRCCS Istituto Tumori “Giovanni Paolo II” (Bari, Italy). Due to the unavailability of dual-energy X-ray absorptiometry (DEXA), bioimpedance analysis (BIA) was performed using the Akern BIA 101 device (Akern S.r.l., Florence, Italy) [42]. Fat-free mass (FFM), fat mass (FM), and phase angle were derived according to clinical guidelines. To reduce hydration-related variability, participants fasted for ≥8 h, avoided caffeine and alcohol for 12 h, and refrained from moderate-to-vigorous physical activity for 24 h before testing. Measurements were taken in the supine position, barefoot, and without metal objects. The same equipment and operators were used at both time points.

Physical performance was evaluated using standardized field tests covering strength, mobility, aerobic capacity, and flexibility. Lower-limb endurance was assessed using the 30-Second Chair Stand Test (30CST), and mobility/balance using the Timed Up-and-Go test (TUG). Upper-limb strength was assessed using Jamar^®^ handgrip dynamometry (GIMA S.p.A., Milan, Italy), and aerobic endurance using the 2-Minute Step Test (TMST). Lower- and upper-body flexibility were assessed using the Chair Sit-and-Reach Test (CSRT) and the Back Scratch Test (BST), respectively. Tests were administered by Adapted Physical Activity (APA) specialists, with 5 min of rest between tests. A familiarization session was conducted one week before baseline to minimize learning effects.

Psychological variables were assessed using standardized self-report questionnaires administered by a clinical psychologist in a private setting two days before physical testing. The battery included the Beck Depression Inventory (BDI), the State-Trait Anxiety Inventory Form Y (STAI-Y1 and STAI-Y2), the Fatigue Severity Scale (FSS), and the European Organization for Research and Treatment of Cancer Quality of Life Questionnaire Core 30 (EORTC QLQ-C30) global health status/HRQoL scale. The psychologist verified completeness of responses, and no assistance was required.

All assessments were repeated at the end of the intervention under the same conditions.

#### 2.3.1. 30-S Chair Stand (30CST) Test

The 30CST test is considered one of the most relevant clinical assessments of functional capacity, as it evaluates lower limb strength and links it to physically demanding daily tasks, such as stair climbing, standing up from a chair or bathtub, or rising from a lying position [43].

The 30CST was administered according to standardized procedures [44,45]. A standard chair without armrests (seat height: 42 cm) was used. Participants started seated upright, feet flat on the floor, and arms crossed over the chest. On the command “1, 2, 3, go”, they repeatedly stood up to full extension and returned to a seated position at a self-selected pace for 30 s. The total number of completed stands within 30 s was recorded as the outcome.

#### 2.3.2. Timed Up-and-Go (TUG) Test

TUG is the most widely used test for evaluating mobility. It measures multiple components of mobility, including static and dynamic balance, gait speed, and lower limb strength [46]. Participants wore their usual footwear and were instructed to perform the test at a self-selected, safe pace; no assistive devices were used. The test began with the participant seated against the backrest of a standard chair (seat height: 42 cm), with arms, facing a line marked 3 m away. At the signal, the participant stood up, walked to the line, turned around, walked back, and sat down again. Timing started on the command and stopped when the participant was fully reseated [47].

#### 2.3.3. Handgrip Strength (HGS) Test

Upper-limb strength was assessed by handgrip strength testing [48]. Grip strength was measured using a mechanical Smedley hand dynamometer (GIMA, Milan, Italy). Participants were seated on a straight-backed chair with feet flat on the floor, shoulder adducted, elbow flexed at 90°, and forearm in a neutral position. They were instructed to squeeze the dynamometer maximally for 5 s. Each hand was tested three times with 30 s rest intervals between trials and a 60 s rest before switching hands; the average value (kg) was used for analysis.

#### 2.3.4. 2-Minute Step Test (TMST)

TMST is designed to evaluate subjective aerobic capacity, which represents a key element of physical fitness (ICC = 0.90) [49]. The target step height was determined as the midpoint between the iliac crest and the patella and marked on the wall. Participants stepped in place for 2 min, raising the knees to the marked height as many times as possible. The score corresponded to the total number of times the right knee reached the target height.

#### 2.3.5. Chair Sit-and-Reach Test (CSRT)

CST is a practical tool to assess hamstring flexibility in older adults. Participants sat on the edge of a standard chair with one leg extended, the heel on the floor, and the ankle dorsiflexed. With the knee kept extended, they slowly reached forward with both hands toward the toes. The distance (cm) between the fingertips and the toes was recorded as the score (positive if beyond the toes, negative if short of the toes) [50].

#### 2.3.6. Back Scratch Test (BST)

Upper-limb (shoulder) flexibility was assessed using the Back Scratch test [45]. Participants reached one hand over the shoulder and the other behind the back, attempting to touch or overlap the middle fingers. The distance (cm) between fingertips was recorded as the score (positive if overlapping, zero if touching, negative if not touching).

#### 2.3.7. Beck Depression Inventory (BDI)

The BDI [51] is a 21-item self-report instrument designed to assess the severity of depressive symptoms over the past four weeks. Each item consists of four statements scored from 0 to 3, with higher total scores indicating greater symptom severity. The scale covers cognitive, affective, and somatic dimensions of depression, including mood, guilt, self-dislike, fatigue, and changes in appetite and sleep. The BDI has demonstrated solid psychometric properties, with Cronbach’s alpha typically ranging from 0.86 to 0.92 in clinical populations [52] and test–retest reliability coefficients above 0.80 [53]. Sensitivity and specificity have been shown to exceed 80% in detecting depressive symptomatology using validated cut-offs.

#### 2.3.8. State-Trait Anxiety Inventory Form Y (STAI-Y)

STAI-Y [54] is a widely validated self-report measure consisting of 40 items, equally divided into two subscales: state anxiety (STAI-Y1), which evaluates transitory anxiety responses to situational stressors, and trait anxiety (STAI-Y2), which assesses the individual’s general predisposition to experience anxiety. Each item is rated on a 4-point Likert scale, with higher scores indicating greater severity of anxiety. The instrument has demonstrated robust psychometric properties. Internal consistency coefficients typically range from 0.89 to 0.95 for the state subscale and from 0.86 to 0.92 for the trait subscale. Test–retest reliability is satisfactory for trait anxiety (r ≈ 0.80) while being lower for state anxiety, as expected due to its situational variability [55].

#### 2.3.9. Fatigue Severity Scale (FSS)

Originally developed to assess fatigue in neurological conditions, the FSS scale [56] has been widely adopted in oncological and chronic disease contexts due to its sensitivity in capturing the functional consequences of fatigue. It consists of 9 statements scored on a 7-point Likert scale, where higher values indicate greater perceived fatigue and interference with daily activities. Participants rate each statement from 1 (“strongly disagree”) to 7 (“strongly agree”), and the total score is calculated as the mean of the 9 items, with higher scores reflecting more severe fatigue. Respondents are instructed to reflect on their experience during the preceding week. The FSS has consistently shown strong psychometric properties across populations. Cronbach’s α typically ranges from 0.88 to 0.93, and test–retest reliability exceeds 0.80, supporting its stability over time [57].

#### 2.3.10. The European Organization for Research and Treatment of Cancer Quality of Life Questionnaire Core 30 (EORTC QLQ-C30)

EORTC QLQ-C30 [58] is a cancer-specific instrument developed to assess HRQoL in oncological populations. It comprises 30 items grouped into five functional domains (physical, role, cognitive, emotional, and social functioning), three symptom scales (fatigue, pain, nausea/vomiting), and a global health status/HRQoL scale. Each item is scored on a 4-point Likert scale, except for the two global health items, which use a 7-point response format. Scores are linearly transformed to a 0–100 scale, with higher scores indicating better functioning or greater symptom burden, depending on the subscale. The QLQ-C30 is one of the most extensively validated instruments in oncology research. It demonstrates high internal consistency (α > 0.70 for most subscales), excellent construct validity, and responsiveness to clinical changes [59].

### 2.4. Exercise Intervention

The exercise sessions were delivered in two small, closely monitored groups under the direct supervision of Adapted Physical Activity (APA) specialists. Training intensity (internal load) was regulated using the Borg Rating of Perceived Exertion (RPE) scale (6–20), recorded after each set of both aerobic and resistance exercises [60]. The target intensity was set at an RPE of 13–15 (moderate). Participants were familiarized with the RPE scale before baseline assessments to support accurate self-reporting. This RPE-based prescription was adopted to standardize intensity across a heterogeneous survivorship sample and to accommodate day-to-day fluctuations in symptoms and tolerance.

Core intervention components (session frequency, structure, target RPE range, and progression principles) were standardized across participants. However, specific exercises, range of motion, and external loads were individually adapted according to clinical history (e.g., mastectomy), treatment-related limitations, and functional capacity, in line with exercise oncology guidelines [7,61].

Progression was implemented by increasing external load (e.g., dumbbell/ankle-weight load), repetitions, or sets for resistance exercises. For aerobics, progression involved increasing task complexity and/or work duration while maintaining the target RPE range (13–15). If RPE was <13, workload was increased in the subsequent session; if RPE was >15, workload was reduced accordingly. These criteria were predefined prior to study initiation and applied consistently throughout the intervention. RPE-based prescription is a validated method to regulate internal load in applied and clinical settings [62] and has been used to support intensity regulation across training modalities in intervention studies [63]. Training fidelity was ensured through continuous direct supervision by APA specialists, real-time correction of exercise technique, verification of adherence to the prescribed RPE range, and systematic recording of attendance.

After baseline assessments, both MTG and ATG followed a 24-week program consisting of two 60 min sessions per week (Mondays and Wednesdays, typically 9:30–10:30 a.m.). The 24-week duration was selected to allow sufficient time for clinically meaningful adaptations in physical and psychological outcomes, consistent with prior exercise oncology trials in cancer survivors [64]. Each session included a 10 min warm-up of brisk walking to raise heart rate and prepare muscles and joints, a 40 min main training phase, and a 10 min cool-down with breathing exercises and static stretching.

#### 2.4.1. Multicomponent Training

Within the multicomponent protocol, the 40 min main phase included cardiorespiratory, flexibility, and resistance components delivered progressively. Aerobic activities consisted of dynamic, rhythmically controlled drills (e.g., jumping jacks, step-ups on a stable surface, alternating standing knee raises, brisk lateral steps, and leg lifts). Intensity was maintained within the target RPE range (13–15). Each aerobic segment ended with ~3 min of light walking to facilitate recovery and transition to the next component. Workload progressively increased throughout the weeks while keeping the target RPE.

Flexibility exercises targeted specific joints and were performed at the maximal pain-free range of motion. The flexibility block included thoracic spine extensions, cat-to-cow sequence, overhead stick reaches, and active internal hip rotations. Exercise duration increased progressively from 30 to 60 s. Each exercise was repeated 1–3 times. Rest intervals of 30–60 s were provided between sets and exercises.

Resistance training targeted major muscle groups and followed progressive overload. Exercises included quadriceps work (e.g., seated leg extensions with ankle weights or half squats with chair assistance), biceps curls (unilateral dumbbell curls), shoulder presses (overhead dumbbell presses), triceps work (dumbbell French presses), pectoral exercises (dumbbell chest press or flyes), and latissimus dorsi engagement (dumbbell rows). At the start of the program, participants completed 1 set of 10–15 repetitions per exercise. The volume progressed gradually up to 3 sets. Load adjustments were guided by the same RPE principle, maintaining perceived effort between 13 and 15. Recovery intervals of 60–120 s were provided between sets and exercises.

To reduce early fatigue and support recovery, an alternating muscle-group approach was used. Upper-body resistance exercises were performed on Mondays, whereas lower-body exercises were performed on Wednesdays. Breathing technique was monitored to avoid breath-holding and limit hemodynamic strain. Upper-body exercises were modified when necessary (e.g., reduced range of motion, unilateral execution, or alternative movements) for participants with prior mastectomy or treatment-related shoulder limitations.

The cool-down included deep breathing and static stretching of major muscle groups. Each stretch was performed to the maximal tolerable limit without joint discomfort. Stretch duration progressed from 10 to 30 s. Stretches were repeated 1–3 times, aiming to accumulate ~60 s per muscle group.

#### 2.4.2. Aerobic Training

In the aerobic protocol, the 40 min main phase consisted of two segments. The first segment included ~25 min of aerobic drills (e.g., jumping jacks, step-ups, high knee raises, lateral steps, and leg lifts). The second segment consisted of ~15 min of continuous walking. Exercise intensity was maintained within the target RPE range (13–15). Progression was implemented by increasing movement complexity and/or duration while keeping perceived exertion within the prescribed RPE range. The goal was to maintain a steady training duration and progressively increase the overall physical demand across the intervention period.

The cool-down mirrored the multicomponent protocol. It included deep breathing exercises and static stretching for all major muscle groups. Stretches were held at the maximal tolerable range without pain for 10–30 s. Each stretch was repeated 1–3 times, for a total of ~60 s per muscle group.

#### 2.4.3. Control Group

Participants allocated to the CG received standard oncological follow-up care as part of their routine oncology follow-up, which typically included medical visits and surveillance but did not include structured or supervised exercise prescriptions; given the heterogeneity of cancer diagnoses, usual care may have varied between participants. Physical activity was monitored using a weekly self-reported physical activity log (activity diary) completed throughout the intervention and reviewed by the study staff. No CG participant reported initiating structured exercise during the trial.

### 2.5. Statistical Analysis

Statistical analyses were performed using SPSS Statistics for Windows, version 26.0 (IBM Corp., Armonk, NY, USA). Normality and homogeneity of variance were assessed using the Shapiro–Wilk and Levene’s tests, respectively. All assumptions for parametric analysis were met, so analyses proceeded accordingly.

To verify the absence of potential confounding factors across experimental conditions, differences in baseline covariates were assessed using one-way analysis of variance (ANOVA). The type of cancer was operationalized as a binary variable (breast cancer vs. other cancers) and used as a covariate in preliminary analyses, as breast cancer represented the largest subgroup, while the other diagnoses were individually rare and heterogeneous.

Intervention effects were tested using a two-way mixed-design ANOVA with Group (MTG, ATG, CG) as the between-subjects factor and Time (pre, post) as the within-subjects factor. The Group × Time interaction was the primary effect of interest. When significant interactions were observed, post hoc analyses were conducted, including within-group pre–post comparisons and between-group comparisons of change scores. Holm–Bonferroni correction was applied within each predefined family of outcomes. Specifically, for multiplicity, control outcomes were grouped by domain: body composition, physical performance, psychological symptoms, and HRQoL. The sample size was planned to detect a moderate Group × Time interaction; therefore, results for individual endpoints were interpreted cautiously.

Effect sizes were reported as partial eta squared (η^2^p) for ANOVA effects and Cohen’s d for post hoc comparisons, interpreted using conventional thresholds. All tests were two-tailed with α = 0.05.

Analyses were conducted on participants with complete baseline and post-intervention data (complete-case; per-protocol). Four participants withdrew during the intervention and were excluded from the final analysis. No imputation was performed; attrition was low (7.8%) and comparable across groups.

## 3. Results

### 3.1. Socio-Demographic and Anamnestic Characteristics of the Total Sample

No adverse events or deviations occurred during the data collection process. Preliminary analyses revealed no statistically significant intergroup differences regarding age, time elapsed since primary treatment completion, cancer recurrence status, familial cancer history, or diagnosis (all *p* > 0.05). No significant between-group differences were observed at baseline for any primary or secondary outcome measures. This included psychological variables such as depressive symptoms, state and trait anxiety, and fatigue. Motor performance metrics included upper and lower extremity strength, balance, and functional mobility. Body composition parameters included fat mass, fat-free mass, phase angle and brachial circumference. Health-related quality of life indices were also assessed (*p* > 0.05 for all variables). Descriptive statistics and baseline group comparisons for all covariates and outcome measures are reported in Table 1 and Table 2.

### 3.2. Effects of the Intervention on Body Composition Parameters

Significant Group × Time interaction effects were observed for lean mass (F(2,44) = 5.957, *p* = 0.005, η^2^_p_ = 0.213), fat mass (F(2,44) = 4.547, *p* = 0.016, η^2^_p_ = 0.171), and arm circumference (F(2,44) = 9.850, *p* < 0.001, η^2^_p_ = 0.309), whereas no significant interaction emerged for phase angle (*p* > 0.05).

Post hoc within-group analyses revealed a significant pre-to-post increase in lean mass in the multicomponent training group (t = −3.325, *p* = 0.027, d = −0.312), with no significant changes in the aerobic training or control groups. Fat mass significantly decreased from pre- to post-intervention in both the multicomponent training group (t = 3.932, *p* = 0.005, d = 0.323) and the aerobic training group (t = 3.220, *p* = 0.034, d = 0.265) while remaining unchanged in the control group. Arm circumference significantly improved only in the multicomponent training group (t = 5.954, *p* < 0.001, d = 0.814), with no significant pre–post differences in the aerobic or control groups.

No significant between-group differences were detected for any body composition parameter (all *p* > 0.05) (Table 3).

### 3.3. Effects of the Intervention on Physical Parameters

A two-way repeated measures ANOVA revealed significant Group × Time interaction effects across all physical performance variables, indicating that the magnitude of change over time differed significantly between groups. Specifically, interaction effects were significant for lower-limb strength (30CST: F(2,44) = 57.97, *p* < 0.001, η^2^_p_ = 0.725), functional mobility (TUG: F(2,44) = 8.22, *p* < 0.001, η^2^_p_ = 0.272), lower and upper-limb flexibility (Sit-and-Reach: F(2,44) = 17.50, *p* < 0.001, η^2^_p_ = 0.443; Back Scratch: F(2,44) = 16.105, *p* < 0.001, η^2^_p_ = 0.423), upper-limb strength (HGS: F(2,44) = 43.61, *p* < 0.001, η^2^_p_ = 0.665), and aerobic endurance (TMST: F(2,44) = 42.74, *p* = 0.003, η^2^_p_ = 0.660).

Post hoc within-group comparisons showed that the MTG improved from pre- to post-intervention in all physical performance outcomes, including 30CST (t = −7.195, *p* < 0.001, d = −2.586), TUG (t = 4.189, *p* < 0.002, d = 0.973, TMST (t = −12.516, *p* < 0.001, d = −2.020), HGS (t = −10.621, *p* < 0.001, d = −1.899), Sit-and-Reach (t = −7.568, *p* < 0.001, d = −1.646), and Back Scratch (t = −6.526, *p* < 0.001, d = −0.886).

No significant within-group differences between pre- and post-intervention were observed in the ATG for 30CST (t = −1.376, *p* = 0.797), TUG (t = 2.335, *p* = 0.266), HGS (t = −0.666, *p* = 0.99), Sit-and-Reach (t = −3.399, *p* = 0.022), or Back Scratch (t = −1.330, *p* = 0.099). Only aerobic endurance improved significantly in the ATG (TMST: t = −8.976, *p* < 0.001, d = −1.449). The CG did not show significant changes in any variable (all *p* > 0.05) (Table 4).

Between-group post hoc comparisons of change scores revealed that the MTG showed greater improvements than the ATG in 30CST (t = 2.053, *p* = 0.046, d = 0.670), Sit-and-Reach (t = 2.338, *p* =0.048, d = 0.744), and HGS (t = 2.333, *p* = 0.049, d = 0.770). These differences between groups were outcome-specific and do not indicate a generalized superiority of one intervention over another across all physical domains. Furthermore, the MTG showed greater improvements than the CG in all physical outcomes except upper limb flexibility (t = 1.216, *p* = 0.461, d = 0.420), including in 30CST (t = 4.387, *p* < 0.001, d = 1.46), TUG (t = −2.557, *p* =0.042, d = −0.84), TMST (t = 4.726, *p* < 0.001, d = 1.608), HGS (t = 2.894, *p* =0.018, d = 0.971), and Sit-and-Reach (t = 3.603, *p* =0.002, d = 1.166) (Table 4).

The ATG showed greater improvements than the CG in 30CST (t = 2.368, *p* = 0.045, d = 0.78), TUG (t = −2.353, *p* = 0.046, d = −0.749), and TMST (t = −0.073, *p* < 0.001, d = 1.386), while no significant differences emerged between these groups for Handgrip Strength, Sit-and-Reach, or Back Scratch flexibility (all *p* > 0.05) (Table 4).

### 3.4. Effects of the Intervention on Psychological Parameters

Significant Group × Time interaction effects were found for all psychological outcomes, indicating differential changes from pre- to post-intervention. These effects reached statistical significance for FSS (F = 6.88, *p* = 0.003, η^2^_p_ = 0.238), STAI-Y1 (F = 20.30, *p* < 0.001, η^2^_p_ = 0.480), STAI-Y2 (F = 27.92, *p* < 0.001, η^2^_p_ = 0.559), and BDI (F = 9.044, *p* < 0.001, η^2^_p_ = 0.291).

For health-related quality of life (EORTC QLQ-C30), Group × Time interaction effects were significant for self-perceived global health (F = 8.851, *p* = 0.003, η^2^_p_ = 0.287), perceived role function (F = 5.983, *p* = 0.005, η^2^_p_ = 0.214), perceived cognitive function (F = 5.049, *p* = 0.011, η^2^_p_ = 0.187), perceived emotional function (F = 19.843, *p* < 0.001, η^2^_p_ = 0.474), and perceived social function (F = 8.550, *p* = 0.003, η^2^_p_ = 0.280), whereas physical functioning did not reach statistical significance (F = 3.106, *p* = 0.055, η^2^_p_ = 0.124). Overall, effect sizes varied across outcomes, with the largest effects observed for anxiety measures and perceived emotional function.

Post hoc pairwise comparisons of pre–post intervention changes showed that both the MTG and ATG had statistically significant improvements in all outcomes of psychological variables (see Table 5).

Post hoc analyses revealed that participants in the MTG experienced greater pre-to-post reductions in FSS scores compared to those in the ATG (t = −2.659, *p* = 0.018, d = −0.739) and CG (t = −5.359, *p* < 0.001, d = −1.515). The ATG also showed a significant reduction in FSS scores compared to the CG (t = −2.743, *p* = 0.018, d = −0.775). These findings refer to specific outcomes and should be interpreted in the context of the significant Group × Time interaction, rather than as evidence of overall superiority of the multicomponent intervention.

For STAI-Y1, the MTG showed greater pre-to-post improvements than the CG (t = −4.449, *p* < 0.001, d = −1.378) and the ATG (t = −5.344, *p* = 0.048, d = −0.621). The ATG also improved significantly compared to the CG (t = −2.666, *p* = 0.037, d = −0.757).

For STAI-Y2, a significant difference was observed between the MTG and CG (t = −4.282, *p* < 0.001, d = −1.404), while no significant difference emerged between the MTG and ATG (t = −0.450, *p* = 0.655). The ATG showed a greater reduction compared to the CG (t = −3.840, *p* < 0.001, d = −1.259).

BDI scores decreased significantly more in the MTG than in both the CG (t = −4.594, *p* < 0.001, d = −1.478) and the ATG (t = −2.355, *p* = 0.046, d = −0.745). The ATG showed a greater reduction than the CG (t = −2.278, *p* = 0.046, d = −0.733).

Regarding self-perceived HRQoL, significant pre-to-post improvements were observed in both the MTG and ATG across multiple QLQ-C30 functioning domains (see Table 5), while CG showed no changes (all *p* > 0.05).

Between-group comparisons at post-test indicated that the MTG showed higher scores than the ATG and CG for self-perceived global health, perceived role function, perceived cognitive function, perceived emotional function, and perceived social function (all *p* < 0.05 for the reported pairwise tests), and the ATG showed higher scores than the CG across domains (Table 5). Although several between-group differences reached statistical significance, these results should be interpreted cautiously given the number of secondary endpoints and the exploratory nature of post hoc comparisons.

## 4. Discussion

### 4.1. Summary of Main Findings

This randomized controlled trial examined the effects of a 24-week supervised MCT program and a comparable volume AT program, compared to a no-exercise control condition, on body composition (fat mass, lean mass, arm circumference), physical performance (strength, mobility, aerobic endurance, and flexibility), psychological symptoms (fatigue, anxiety, and depression), and health-related quality of life in cancer survivors.

Overall, both exercise programs were accompanied by improvements in selected functional and psychological domains, while the control condition did not show a comparable pattern of change. Improvements in physical performance, psychological symptoms, and quality of life were observed following both exercise programs, whereas AT training was primarily associated with increases in endurance and reductions in fat mass. MCT was associated with larger improvements in selected functional and patient-reported outcomes. These findings are consistent with current exercise oncology recommendations that support structured exercise as a fundamental component of survivor care [7,61,65].

### 4.2. Interpretation by Outcome Domain

#### 4.2.1. Body Composition

The results indicate that the exercise interventions were associated with within-program changes in selected body composition parameters, especially in lean mass, fat mass, and arm circumference. Participants in the multicomponent group showed an increase in both lean mass and arm circumference. This was accompanied by a reduction in fat mass, suggesting a concurrent pattern of increased lean-related indices and reduced adiposity within that program.

These data are consistent with the literature on the effects of physical exercise in cancer survivors. This population often shows metabolic alterations and reduced anabolic response after cancer treatments such as chemotherapy, radiotherapy, and hormone therapies. Endocrine imbalances related to the disease also contribute to the condition [27]. These factors explain why changes in BMI are often minimal despite structured training programs [66,67].

The pattern observed in the MCT program is consistent with meta-analyses, which indicate the relevance of resistance-oriented stimuli for lean tissue maintenance and gains [68]. Recent systematic reviews have shown that multicomponent interventions are effective for improving both lean body mass and bone mass in various populations [69]. A randomized controlled trial found significant improvements in body composition after an 8-month multicomponent program. This is consistent with the utility of this approach for improving compositional outcomes [70]. Previous meta-analyses have also shown that the volume and frequency of resistance training independently contribute to gains in lean mass, with higher frequencies associated with greater hypertrophic results [68].

An increase in lean mass was observed. Loss of skeletal muscle tissue is common after cancer treatment and is linked to reduced strength, lower quality of life, and higher mortality [71]. However, as the literature highlights, the extent of these gains in patients tends to be less than in healthy or elderly non-cancer populations. This is probably due to “anabolic resistance”, which limits protein synthesis and muscle recovery [72].

The aerobic training group experienced a reduction in fat mass, consistent with evidence that aerobic training increases lipid oxidation and energy expenditure [73]. This type of training is associated with improved body composition, as evidenced by increased fat utilization [74]. However, since lean mass did not increase significantly, aerobic training alone may provide limited stimulus for muscle hypertrophy, highlighting the role of resistance components in programs aimed at maintaining or growing muscle mass.

Despite within-group changes, clear between-group differences were not observed in body composition parameters. This may reflect the multifactorial nature of bodily adaptations and individual variability in response to training [75], as well as the relatively short procedure duration.

The absence of significant changes in phase angle, despite changes in body composition, could be due to the limited sensitivity of this indicator to short-term changes. Another reason could be the need for longer protocols. Phase angle, measured by bioelectrical impedance analysis, is considered a marker of cellular health and membrane integrity [76].

Recent studies indicate that variations in this parameter require longer interventions, especially in those with already optimal baseline values [77]. Resistance training has been shown to counteract the negative impact of aging on cellular function and integrity [78]. Overall, these findings support the feasibility of structured exercise in cancer survivorship and indicate that including resistance elements may be relevant when the clinical goal includes lean mass maintenance alongside reductions in adiposity, while acknowledging that longer follow-up and dietary contextualization are needed to strengthen inferences in body composition adaptations, a key goal for metabolic, functional health, and long-term prognosis in survivorship care.

#### 4.2.2. Physical Performance

Structured exercise interventions can significantly enhance multiple domains of physical performance in cancer survivors [7,61]. Regarding lower-limb strength, as measured by the 30 s Chair Stand Test, post hoc between-group comparisons indicated greater improvement in the MTG than in the ATG and CG, while changes in the ATG were not statistically significant. These findings strengthen the rationale that resistance-based interventions might enhance clinical populations’ sit-to-stand performance [79]. This supports multicomponent protocols combining resistance, balance, and functional exercises to promote lower-limb strength and its transfer to daily function [80,81].

Functional mobility showed a statistically significant pre–post change in the MTG, whereas the ATG did not show a statistically significant pre–post change.

The observed pattern may reflect the program’s emphasis on dynamic balance challenges, multi-directional movements, and functional task practice. These elements directly translate to improved mobility performance [82]. Concerning aerobic endurance capacity, both exercise groups showed statistically significant improvements relative to CG. This is consistent with the established cardiorespiratory benefits of aerobic-oriented exercise exposure in cancer survivors [8,83].

Between-group differences may reflect the combined metabolic and neuromuscular demands of multicomponent sessions, supporting concurrent adaptations in endurance and exercise tolerance [84,85].

Upper-limb strength, assessed through handgrip strength testing, showed a statistically significant pre–post increase in MTG, whereas the ATG did not show a statistically significant pre–post change. Handgrip strength serves as a reliable indicator of overall muscle strength and functional capacity in cancer survivors [86], with lower values associated with reduced quality of life and increased mortality risk [87]. This pattern is consistent with the limited upper-extremity strength stimulus provided by protocols without a dedicated resistance component [82]. The MTG’s resistance training components specifically targeting upper-body musculature were essential for maintaining and improving grip strength [88], which is crucial for activities of daily living in cancer survivors [89]. Flexibility outcomes revealed distinct patterns across the two measures examined. Lower-limb flexibility, evaluated using the Sit-and-Reach test, showed greater improvement in the MTG, consistent with the inclusion of specific stretching targeting the hip flexors and hamstring muscles [90]. In contrast, upper-limb flexibility assessed through the Back Scratch test did not improve in either of the experimental groups.

These findings emphasize that flexibility gains require targeted stretching interventions [79], as aerobic training and multicomponent provide insufficient range-of-motion stimulus for meaningful improvements in joint mobility [88].

The CG showed no statistically significant changes across outcomes, supporting the interpretation that structured exercise exposure is associated with more favorable trajectories in physical function during survivorship [65]. Overall, the between-group findings suggest that both exercise modalities were associated with improvements in selected domains relative to control, while multicomponent training showed larger changes than aerobic training for specific functional outcomes [91].

#### 4.2.3. Psychological Symptoms

Both exercise interventions were associated with modest reductions from baseline to post-intervention in anxiety and depression-related outcomes, consistent with structured exercise as a non-pharmacological component of supportive care in cancer survivorship [92]. These changes are interpreted primarily in terms of their potential to alleviate overall symptom burden during survivorship, rather than as evidence of large shifts in psychological status.

Exercise-related improvements in affective symptoms have been discussed in the literature in relation to broad stress- and recovery-related pathways, including neuroendocrine and immune modulation [93,94].

In our trial, between-group comparisons indicated greater reductions in depressive symptoms in the MTG than in the ATG, suggesting a potential advantage of integrating multiple training components for depression-related symptom burden compared with a single-component protocol.

A plausible interpretation is that the resistance component may support concurrent gains in strength and body composition [71], which can be salient for survivors coping with treatment-related physical changes. Improvements in lean mass and physical capacity may align with more favorable body image and self-perception [95,96], and may support perceived self-efficacy, which has been associated with lower depressive symptom scores in survivorship [97]. In addition, MCT provides varied stimuli, which may enhance intrinsic motivation, adherence, and enjoyment, factors commonly linked to the maintenance of psychological benefits over time [98].

Trait anxiety showed reductions over the intervention period, while no significant differences emerged between the two training protocols. This finding involves conceptualizing trait anxiety as a relatively stable personality disposition, less susceptible to modulation by specific characteristics of an intervention over short periods [54]. In contrast to state anxiety, which reflects transient fluctuations in response to situational demands, trait anxiety involves enduring patterns of affective reactivity and cognitive appraisal that may respond to regular physical activity through generalized psychophysiological adaptations [99] but are less influenced by the type or complexity of exercise modality [100,101]. Both MCT and AT may have contributed to lower trait anxiety through shared, non-modality-specific psychophysiological adaptations discussed in the literature, e.g., hypothalamus–pituitary–adrenal axis (HPA), modulation, monoaminergic tone, and brain-derived neurotrophic factor (BDNF) expression [31,102,103].

Fatigue, a major side effect in survivors that significantly impairs HRQoL [104], showed statistically significant reductions in both exercise groups relative to CG, with between-group comparisons indicating larger reductions in MTG than in ATG and CG. Although the causes of fatigue are likely multifactorial, low physical activity levels, decreased muscle mass, and impaired fitness have been identified as potential contributors [15,105,106]. Improvements in strength and cardiorespiratory capacity enhance functional capacity and muscle mass while reducing fatigue [28,107]. Combined aerobic and resistance training may improve lean mass and mitochondrial efficiency, reducing perceived exertion during daily activities and lowering fatigue severity, with potential implications for everyday tolerance and functioning [108,109].

The reduction in fatigue observed in MTG is consistent with the previous limited evidence that combining aerobic and resistance exercise prescriptions is among the most effective approaches for fatigue [110]. Physiologically, resistance blocks in the MCT regimen may facilitate restoration of lean mass and mitochondrial density, reducing the energy cost of daily activity and peripheral fatigue signals [111,112].

#### 4.2.4. Health-Related Quality of Life

Cancer-related treatments can compromise HRQoL through converging physical and psychological sequelae (e.g., fatigue, deconditioning, and mood symptoms), and structured exercise is increasingly recognized as a relevant supportive strategy [113,114].

Previous studies have shown that a regular exercise program applied to patients during or after cancer treatments improved HRQoL alongside physical fitness, fatigue, and psychosocial well-being [33,115].

It has been observed that depression and anxiety are two of the factors that influence HRQoL, and both tend to improve concurrently [116]. According to the EORTC QLQ-C30 assessments, a study by Canário et al. (2016) [117] found that active subjects had higher scores on all scales, and their HRQoL scores were better than those of the non-active group.

In our study, between-group comparisons further indicated larger gains in several HRQoL domains in the MTG than in the ATG and CG, suggesting that MCT may be advantageous for several patient-reported outcomes. A combined resistance and aerobic training program, similar to the one in our study, applied to elderly breast cancer survivors showed significant improvements in quality-of-life parameters, including physical function, health, pain, general sense of health, vitality, social functioning, and fatigue [118].

Improvements from exercise extend across perceived cognitive, social, and emotional domains for cancer survivors. Improvements in muscle strength, flexibility, and aerobic capacity may support perceived role functioning and daily independence and health perception [119,120,121].

MCT may foster a heightened subjective sense of mental engagement due to the variety and complexity of the activities involved. This may be reflected in greater perceived attentional involvement during sessions [122]. Social benefits may also emerge at the level of perceived social function, especially in group-based exercise settings. Participation in shared activities can enhance feelings of social connection, belonging, and support, thereby reducing perceived social isolation and positively influencing self-reported social well-being [123,124]. Emotionally, engaging in regular MCT may strengthen subjective feelings of competence, confidence, and body acceptance, which are closely linked to improved emotional well-being as reported by participants [125].

#### 4.2.5. Strengths and Limitations

Several methodological features strengthen the interpretability of our results. The randomized, controlled, parallel-group design, the equivalent-volume comparison between MCT and AT, and the inclusion of a non-exercise control group were intended to isolate training-related effects from time-related changes. The multidimensional outcome set (body composition, functional performance, psychological symptoms, and HRQoL) reflects the clinical complexity of survivorship beyond single-endpoint approaches. Internal validity was supported by supervised delivery in small groups, high adherence, and standardized assessments (controlled environment, fixed morning schedule, and pre-baseline familiarization). Allocation concealment and blinding of outcome assessors and data analysts further reduced detection and analysis bias.

These strengths should be interpreted alongside several limitations. The relatively small sample size limited statistical power and precision of effect estimate and precluded adequately powered subgroup analyses (e.g., by cancer type or treatment history). Psychological and HRQoL outcomes relied on self-report measures, which remain susceptible to reporting bias; item-level data were unavailable to re-estimate internal consistency within the present sample. Participants presented a relatively preserved baseline physical status, which may limit generalizability and restrict detectable change (ceiling effects). The intervention dose (two supervised sessions/week) and the use of RPE-based intensity prescription, rather than percentage of maximum heart rate (%HRmax) or percentage of one-repetition maximum (%1 RM), may limit comparability with trials using externally anchored prescriptions and could have attenuated between-group differentiation. The 24-week duration without post-intervention follow-up prevents conclusions regarding the durability of effects.

Diagnostic and treatment heterogeneity represents an additional limitation: while most participants were breast cancer survivors, other cancer types were included to increase sample size and generalizability. This heterogeneity may dilute tumor- or treatment-specific effects, particularly for outcomes sensitive to endocrine therapy, cytotoxic regimens, or disease course. Cancer stage was not available for all participants and could not be examined as a moderator. Although physical activity was monitored via weekly self-report logs, the absence of device-based monitoring means that unreported changes in habitual activity cannot be completely excluded.

Although multiplicity was addressed using Holm–Bonferroni correction within outcome domains, multiple secondary endpoints warrant cautious interpretation of exploratory post hoc findings. Analyses were conducted per protocol; despite low attrition, the absence of intention-to-treat analysis may introduce attrition bias.

Finally, diet and pharmacological therapy were not systematically monitored during the intervention period, and no nutritional intervention was provided. This limits the interpretation of body composition changes, given the influence of energy balance, protein availability, and hydration status on BIA-derived indices (e.g., lean mass and phase angle). In addition, although participants were instructed to maintain their prescribed medications, unrecorded changes in pharmacological therapy (e.g., dose adjustments or initiation/discontinuation of symptomatic or psychotropic treatments) cannot be excluded and may have contributed to variability in patient-reported outcomes across the study period.

## 5. Conclusions

The findings suggest that a 24-week MCT program is associated with meaningful improvements in physical performance, psychological well-being, and health-related quality of life in adults who have completed cancer treatment, with changes observed in several outcomes compared with the AT and control conditions. MCT was associated with larger improvements in selected outcomes, including strength, flexibility, mobility, fatigue, anxiety, depression, and multiple functional domains of HRQoL, whereas other outcomes showed comparable changes between exercise modalities. The absence of adverse effects confirms the safety and tolerability of the intervention. These improvements should be interpreted as specific to the outcomes measured and this sample, rather than indicative of generalized superiority across all domains. Given these findings, healthcare providers and policymakers may consider implementing multicomponent exercise programs in cancer rehabilitation, particularly when targeting multiple functional outcomes. Further studies with larger samples remain necessary to consolidate the evidence for sustained long-term benefits.

## Figures and Tables

**Table 1:** Sociodemographic and clinical characteristics of the entire sample and comparison between groups.

Covariate	Total Sample (*n* = 47)	MTG (*n* = 15)	ATG (*n* = 16)	CG (*n* = 16)	*p*-Value
Age (Years), Mean ± SD	63.04 ± 8.91	67.1 ± 7.1	60.9 ± 7.8	60.9 ± 10.5	0.08 ^a^
Sex, n (%)					
Male	11 (23.4)	2 (12.5)	5 (31.2)	4 (26.7)	
Female	36 (76.6)	14 (87.5)	11 (68.8)	11 (73.3)	0.43 ^b^
Marital status, n (%)					
Married	38 (80.9)	12 (75.0)	15 (93.8)	11 (73.0)	
Unmarried	9 (19.2)	4 (25.0)	1 (6.2)	4 (26.7)	0.27 ^b^
Cancer type, n (%)					
Breast cancer	31 (66.0)	10 (62.5)	10 (62.5)	11 (73.3)	
Other cancer types	16 (34.0)	6 (37.5)	6 (37.5)	4 (26.7)	0.77 ^b^
Non-Hodgkin lymphoma	5 (10.6)				
Prostate cancer	2 (4.3)				
Leukemia	2 (4.3)				
Ductal cancer	1 (2.1)				
Endometrial cancer	1 (2.1)				
Liver cancer	1 (2.1)				
Intestinal cancer	1 (2.1)				
Pancreatic cancer	1 (2.1)				
Renal cancer	1 (2.1)				
Gastric cancer	1 (2.1)				
Time since treatment in months (Mean ± SD)	17.40 ± 9.41	20.2 ± 9.7	17.5 ± 10.2	14.2 ± 7.7	0.21 ^a^
Cancer recurrence status, n (%)					
Yes	6 (12.8)	2 (12.5)	2 (12.5)	2 (13.3)	0.99 ^b^
No	41 (87.2)	14 (87.5)	14 (87.5)	13 (86.7)	
Cancer familiarity, n (%)					
Yes	23 (48.9)	8 (50.0)	7 (43.8)	8 (53.3)	0.86 ^b^
No	24 (51.1)	8 (50.0)	9 (56.2)	7 (46.7)	

Abbreviations: SD = standard deviation; ANOVA = one-way analysis of variance; *p*-value = probability value. Notes. ^a^ One-way ANOVA for continuous variables; ^b^ Chi-square test for categorical variables; Fisher’s exact test used when expected counts were <5.

**Table 2:** Baseline comparison of outcome measures across groups.

Variables	MTG (Mean ± SD)	ATG (Mean ± SD)	CG (Mean ± SD)	F	*p*
Lean Mass (kg)	48.36 (±6.21)	51.13 (±7.78)	48.97 (±5.56)	0.774	0.46
Fat Mass (kg)	24.60 (±7.72)	22.51 (±8.23)	23.26 (±8.34)	0.273	0.76
Arm circumference (cm)	30.75 (±3.71)	30.81 (±3.72)	30.73 (±3.01)	0.002	0.99
Phase angle (°)	6.18 (±1.21)	6.48 (±1.09)	6.26 (±1.00)	0.324	0.72
30- CST (n)	10.50 (±3.67)	11.81 (±2.63)	10.33 (±2.02)	1.252	0.29
TUG (s)	9.32 (±2.94)	8.96 (±1.15)	9.67 (±3.62)	0.253	0.77
CSRT (cm)	−10.25 (±9.97)	−9.93 (±7.91)	−13.26 (±11.93)	0.515	0.60
BST (cm)	−8.56 (±5.30)	−9.93 (±9.83)	−7.66 (±8.22)	0.318	0.72
HGS (kg)	21.25 (±7.76)	22.10 (±8.30)	22.39 (±7.01)	0.093	0.91
TMST (n)	58.87 (±17.67)	59.93 (±20.00)	49.40 (±10.46)	1.857	0.16
FSS	31.88 (±10.19)	39.12 (±13.49)	40.06 (±12.69)	2.444	0.09
STAI-Y1	35.50 (±5.70)	43.37 (±8.29)	40.26 (±11.84)	3.177	0.051
STAI-Y2	49.0 (±8.87)	54.18 (±11.90)	56.06 (±13.31)	1.595	0.21
BDI	17.81 (±7.80)	26.62 (±12.37)	27.66 (±15.05)	3.186	0.051
EORTC QLQ-C30	56.81 (±18.07)	51.06 (±19.80)	47.80 (±21.75)	0.82	0.44

Notes. Data are presented as mean ± standard deviation (SD). Between-group comparisons of all outcome variables at baseline were conducted using one-way ANOVA. No significant between-group differences were found for any outcome measure at baseline (all *p* > 0.05); MTG = Multicomponent Training Group; ATG = Aerobic Training Group; CG = Control Group; CST, Chair Stand Test; TUG, Timed Up and Go; CSRT, Chair Sit-and-Reach Test; BST, Back Scratch Test; HGS, Handgrip Strength; TMST, 2-Minute Step Test; FSS, Fatigue Severity Scale; STAI-Y1, State-Trait Anxiety Inventory; STAI-Y2, State-Trait Anxiety Inventory; BDI, Beck Depression Inventory; kg, kilograms; cm, centimeters; °, degrees; n, number; s, seconds.

**Table 3:** Changes after 24 weeks in body composition.

	MTG (*n* = 16)		ATG (*n* = 16)		CG (*n* = 15)	
Variables	Pre-Test	Post-Test	Pre-Test	Post-Test	Pre-Test	Post-Test
Body Composition						
Lean Mass (kg)	48.369 (±6.211)	50.444 (±6.622) *	51.138 (±7.782)	50.388 (±7.745)	48.973 (±5.566)	48.627 (±5.477)
Fat Mass (kg)	24.600 (±7.726)	22.019 (±6.719) *	22.512 (±8.231)	20.394 (±8.069) *	23.260 (±8.341)	23.360 (±8.849)
Arm circumference (cm)	30.750 (±3.715)	28.063 (±2.516) *	30.813 (±3.728)	30.313 (±3.554)	30.733 (±3.011)	30.733 (±3.081)
Phase angle (°)	6.181 (±1.211)	6.075 (±0.922)	6.487 (±1.097)	6.275 (±0.609)	6.267 (±1.002)	6.220 (±0.701)

Notes. Data are expressed as mean (±SD). * Statistically significant difference within groups from pre- to post-intervention (Holm–Bonferroni test, *p* < 0.05). MTG = Multicomponent Training Group; ATG = Aerobic Training Group; CG = Control Group; kg, kilograms; cm, centimeters; °, degrees.

**Table 4:** Changes after 24 weeks in physical parameters.

	MTG (*n* = 16)		ATG (*n* = 16)		CG (*n* = 15)	
Variables	Pre-Test	Post-Test	Pre-Test	Post-Test	Pre-Test	Post-Test
Physical Parameters						
30 CST (n)	10.500 (±3.670)	18.063 (±4.725) *#	11.813 (±2.639)	12.625 (±2.604)	10.333 (±2.024)	9.267 (±1.534)
TUG (s)	9.324 (±2.943)	**7.031 (±1.524)** *	8.969 (±1.156)	7.691 (±1.818)	9.673 (±3.625)	10.516 (±2.241)
CSRT (n)	−10.250 (±9.970)	3.438 (±1.922) *#	−9.938 (±7.912)	−9.250 (±3.587)	−13.267 (±11.931)	−12.933 (±10.039)
BST (cm)	−8.563 (±5.304)	−2.125 (2.655) *	−9.938 (±9.835)	−8.625 (±8.453)	−7.667 (±8.226)	−9.133 (±6.823)
HGS (kg)	21.255 (±7.767)	34.784 (± 5.396) *#	22.107 (±8.303)	22.955 (±7.634)	22.398 (±7.012)	19.801 (±6.151)
TMST (n)	58.875 (±17.671)	92.688 (± 19.189) *	59.938 (±20.008)	84.188 (±18.988) *	49.400 (±10.466)	48.333 (±10.383)

Notes. Data are expressed as mean (±SD). * Statistically significant difference within groups from pre- to post-intervention (Holm–Bonferroni, *p* < 0.05). # Statistically significant difference between the MTG and ATG group (Holm–Bonferroni, *p* < 0.05); MTG = Multicomponent Training Group; ATG = Aerobic Training Group; CG = Control Group; CST, Chair Stand Test; TUG, Timed Up and Go; CSRT, Chair Sit-and-Reach Test; BST, Back Scratch Test; HGS, Handgrip Strength; TMST, 2-Minute Step Test; kg, kilograms; cm, centimeters; n, number; s, seconds.

**Table 5:** Changes after 24 weeks in psychological parameters.

	MTG (*n* = 16)		ATG (*n* = 16)		CG (*n* = 15)	
Variables	Pre-Test	Post-Test	Pre-Test	Post-Test	Pre-Test	Post-Test
Psychological Parameters						
FSS	31.188 (±10.913)	11.750 (±3.550) *#	39.125 (±12.696)	20.375 (±10.683) *	40.067 (±13.493)	36.800 (±13.056)
STAI-Y1	35.500 (±5.704)	24.375 (±6.820) *#	43.375 (±8.294)	27.188 (±7.713) *	40.267 (±11.847)	43.333 (±10.168)
STAI-Y2	49.000 (±8.877)	30.688 (7.012) *	54.188 (±11.901)	28.563 (±7.874) *	56.067 (±13.312)	53.267 (±12.970)
BDI	17.813 (±7.808)	3.875 (±2.500) *#	26.625 (±12.377)	10.750 (±5.905) *	27.667 (±15.051)	25.133 (±14.015)
SELF-PERCEIVED GLOBAL HEALTH	56.813 (±18.075)	91.375 (±8.686) *#	51.063 (±19.804)	74.500 (±8.571) *	47.800 (±21.759)	55.600 (±20.486)
PERCEIVED ROLE FUNCTION	76.875 (±21.808)	98.375 (±2.527) *#	64.625 (±25.879)	83.938 (±11.807) *	53.533 (±25.576)	54.600 (±25.823)
PERCEIVED EMOTIONAL FUNCTION	49.375 (±25.881)	93.875 (±10.236) *#	36.375 (±15.095)	72.625 (±9.818) *	28.867 (±19.643)	36.800 (±27.814)
PERCEIVED COGNITIVE FUNCTION	65.625 (±27.548)	89.688 (±9.300) *#	53.438 (±26.038)	71.750 (±15.395) *	39.000 (±30.270)	38.933 (±29.246)
PERCEIVED SOCIAL FUNCTION	54.188 (±24.868)	97.375 (±7.320) *#	50.188 (±17.766)	83.375 (±16.194) *	49.133 (±14.267)	63.200 (±13.955)

Notes. Data are expressed as mean (±SD). * Statistically significant difference within groups from pre- to post-intervention (Holm–Bonferroni, *p* < 0.05). # Statistically significant difference between the MTG and ATG group (Holm–Bonferroni, *p* < 0.05); MTG = Multicomponent Training Group; ATG = Aerobic Training Group; CG = Control Group; FSS, Fatigue Severity Scale; STAI-Y1, State-Trait Anxiety Inventory; STAI-Y2, State-Trait Anxiety Inventory; BDI, Beck Depression Inventory.

## Data Availability

The data presented in this study are available upon request from the first author. The data are not publicly available due to privacy concerns.
